# Nutrient Clusters Associated with the Dietary Inflammatory Index in Patients with Diabetes and Prediabetes: A Prospective Observational Study

**DOI:** 10.3390/nu18030422

**Published:** 2026-01-27

**Authors:** Jiwon Park, Myoung Soo Kim

**Affiliations:** 1 School of Nursing, University of Maryland Baltimore, Baltimore, MD 21201, USA; 2 Department of Nursing, Pukyong National University, Busan 48513, Republic of Korea

**Keywords:** antioxidants, diabetes mellitus, diet, fatty acids, prediabetic status

## Abstract

**Background/Objectives**: The dietary inflammatory index (DII) has been widely used to examine dietary inflammation in chronic diseases; however, the relative contribution of individual nutrients to the total DII score remains unclear. Identifying nutrient clusters that strongly influence the energy-adjusted DII (E-DII) in patients with diabetes and prediabetes may provide practical guidance for dietary counselling and intervention. This study aimed to identify nutrient clusters based on dietary intake and examine their association with the E-DII in patients with diabetes and prediabetes. **Methods**: In total, 408 dietary records of 17 patients were analysed. The E-DII was calculated from the reported dietary intake using photographs. Exploratory factor analysis was used to derive nutrient clusters, and ordinary logistic regression analysis was applied to examine their association with the E-DII tertiles. **Results**: Five nutrient clusters (antioxidant-mineral, protein-B complex, fatty acids, plant-lipids, and immune-modulating micronutrients) were extracted, explaining 69.3% of the total variance. Ordinary logistic regression showed that antioxidant-mineral, fatty acids, and immune-modulating micronutrients predicted classification between low, intermediate, and high E-DII groups. **Conclusions**: Antioxidant-mineral, fatty acids, and immune-modulating micronutrients were associated with a lower probability of belonging to the pro-inflammatory group. The identification of these clusters highlighted specific nutrient combinations that may protect against diet-induced inflammation. These results provided clinically relevant evidence that nutritional strategies emphasising fruits, vegetables, healthy fats, and balanced protein sources may contribute to lowering dietary inflammatory potential and improving metabolic health in patients with diabetes and prediabetes.

## 1. Introduction

The global prevalence of diabetes is rapidly increasing, with projections estimating that 780 million people will be diagnosed by diabetes by 2045 [1]. In Korea, 13.8% of adults aged ≥30 years had type 2 diabetes mellitus (T2DM) as of 2018, and approximately 44.3% were classified as having prediabetes [2]. Approximately 30% of patients with prediabetes progress to T2DM [3]. Lifestyle modifications, particularly dietary interventions, are essential for preventing disease progression and associated complications [4]. Dietary patterns influence systemic inflammation and glycemic regulation, as reflected by inflammatory biomarkers [5], drawing increasing attention to the inflammatory potential of diet.

The dietary inflammatory index (DII), developed by Cavicchia et al. [6] and refined by Shivappa et al. [7], quantifies the inflammatory potential of an individual’s diet based on 45 food parameters and 6 inflammatory biomarkers. The DII has been widely used to explore the association between dietary inflammation and chronic diseases, particularly T2DM and prediabetes. Higher DII scores have been linked to increased risk of prediabetes [8] and T2DM [9], and lower scores are associated with reduced mortality [10] and improved vascular outcomes [11]. Variants such as the energy-adjusted DII (E-DII) have broadened its applicability across diverse populations and health outcomes [12,13]. However, owing to the complexity of the DII scoring method, the way in which individual nutrients contribute to the total score is not intuitively clear. The DII is calculated by converting each nutrient intake into a z-score using the global means and standard deviations from 11 countries, transforming them into percentiles, and multiplying by inflammatory effect scores (−1 to +1), which are then summed. The E-DII adjusts this to 1000 kcal to account for energy intake variations across populations. Due to this weighted calculation, it remains unclear which nutrients should be prioritised or avoided in real-world dietary interventions. Many nutrients are co-consumed in patterns that may have synergistic or antagonistic inflammatory effects [14,15], making isolated nutrient analysis potentially misleading.

Many studies have classified patients into low, intermediate, and high DII groups [16,17]. Notably, patients in the highest tertile of DII scores—indicating proinflammatory dietary patterns—demonstrated a 19-fold increased risk of developing prediabetes over 5 years compared with those in the lowest tertile [18]. High DII scores have also been linked to a 39% greater risk of non-alcoholic fatty liver disease [19] and are independently associated with sarcopenia in patients with T2DM [20]. These findings underscore the clinical significance of dietary inflammation and value of stratifying patients based on their dietary inflammatory profile. Identifying the nutrient groups that most strongly influence the E-DII may offer practical insights for dietary counseling in patients with diabetes or prediabetes. To this end, the present study adopted an exploratory, pattern-based approach to identify nutrient clusters associated with E-DII tertiles and develop a classification model that distinguishes dietary inflammatory profiles across low, intermediate, and high groups. The ultimate goal was to inform targeted nutritional interventions and help prevent progression toward more pro- inflammatory dietary patterns.

## 2. Materials and Methods

### 2.1. Study Design, Population, and Sample Size

This study was a prospective observational study. The participants were recruited from the outpatient departments of internal medicine and surgery at a tertiary hospital. The inclusion criteria were as follows: (1) T2DM or prediabetes, (2) agreement to continuous glucose monitoring for 4 weeks, and (3) provision of dietary information at least 10 times during the study period. The exclusion criteria were as follows: (a) body mass index (BMI) < 25 kg/m^2^, (b) not consuming hypoglycemic agents, and (c) missing anthropometric or hemodynamic data.

In total, 23 participants were initially enrolled. Of these, 19 met the inclusion and exclusion criteria. The participants were instructed to provide photographs of their meals before and after eating. The data from 17 participants were included in the final analysis. Two were excluded because of poor photo quality or an insufficient number of meal images. Over 4 weeks, with 3 meals per day, approximately 60 dietary records were expected per participant. Using 24 entries as the minimum threshold, 24 meal photographs were randomly extracted from each participant. There were no missing values among the variables included in the final analysis. This process yielded 408 meal-based observations. However, although not an absolute standard, a sample size of 408 for 25 nutrient variables was considered relatively stable for factor analysis. This aligns with recommendations suggesting at least 300 total observations or a subject-to-variable ratio of 10:1 to ensure stable factor solutions [21].

### 2.2. Measures

The original DII includes 45 food parameters, including macronutrients, micronutrients, spices, garlic, onions, and other food components. In the present study, spices, garlic, onions, and alcohol were excluded because of a high proportion of missing data. The final analysis included the following nutrients: carbohydrates; protein; vegetable lipids; animal lipids; dietary fiber; cholesterol; saturated fatty acids (SFA); monounsaturated fatty acids (MUFA); omega-3 fatty acids (n-3 PUFA); omega-6 fatty acids (n-6 PUFA); Vitamin B1 (thiamin); Vitamin B2 (riboflavin); Vitamin B3 (niacin); Vitamin B6 (pyridoxine); Vitamin B9 (folic acid); Vitamin B12 (cobalamin); Vitamins A, C, D, and E; beta-carotene; iron; magnesium; selenium; and zinc.

The E-DII was calculated to reflect the nutrient density and normalize the nutrient intake to 1000 kcal. Z-scores were computed based on the global mean and standard deviation following the method established by Shivappa et al. [7], and scoring was applied according to Hébert et al. [22]. In the present study, the total daily E-DII scores were computed by adjusting the nutrient intake for each meal (breakfast, lunch, and dinner) and multiplying by three to approximate the intake per 1000 kcal for three daily meals. Although this approach differs from the conventional daily based E-DII calculation, it was adopted to accommodate the meal-based photograph data collection method used in this study. As the E-DII is a composite index derived from literature-based inflammatory weights of individual nutrients, the present analysis did not aim to establish independent prediction but rather to explore how empirically derived nutrient intake patterns were associated with graded levels of dietary inflammatory potential.

### 2.3. Data Collection

A dietary intake survey was conducted using a 24 h photo estimation method [23]. Participants were instructed to take photographs of their meals before and after consumption and provide them to trained dietary investigators. To improve the measurement accuracy, standardised food plates were distributed to all participants. In addition to the photographs, the participants were asked to provide the names of the foods and cooking methods via text messages. Labelled packaging photographs, including product names and nutritional information, were recommended for commercially available products. Nutrient intake was analysed using the Computer Aided Nutritional Analysis Program (version 5.0) (CAN-Pro 5.0; The Korean Nutrition Society, Seoul, Republic of Korea). Investigators searched for the corresponding foods in the database, selected the appropriate entries, and recorded information such as portion size and nutrient composition. Based on the entries, three trained investigators estimated the nutrient content per serving and recorded the data. The food database of the program was standardised to reflect one serving per individual, and when necessary, the total intake was adjusted proportionally based on visual estimation.

In cases where participants dined at restaurants, the investigators referred to the nutritional information of comparable dishes in the CAN-Pro database to improve accuracy. Before data entry, the investigators received training in dietary evaluation and meal planning to enhance consistency. To ensure intra- and inter-rater reliability, each investigator independently assessed the same dietary entries. Intra-rater reliability was analysed using the intraclass correlation coefficient (ICC), with ICC values for carbohydrate, lipid, protein, and dietary fibre intake exceeding 0.85. According to the criteria of Koo and Mae [24], an ICC between 0.75 and 0.90 indicates good reliability.

### 2.4. Ethical Considerations

Before the investigation commenced, all participants were fully informed about the study objectives, procedures, data confidentiality, and the voluntary nature of their involvement. They were assured that their personal information would be kept confidential and used solely for research purposes and that they could withdraw from the study at any point without any consequences. Written informed consent was obtained from all the participants. Ethical approval was obtained from the Institutional Review Board of Pukyong National University (IRB No. 1041386-202211-HR-71-02). This study followed the STROBE reporting guideline for observational research [25] (Supplementary Table S1).

### 2.5. Statistical Analysis

Data were analysed using the Statistical Package for the Social Sciences/Windows 27.0 (IBM Corp., Armonk, NY, USA) and R (version 4.4.0; R Foundation for Statistical Computing, Vienna, Austria). Normality was assessed using both the Kolmogorov–Smirnov and Shapiro–Wilk tests, which revealed that some variables did not meet the assumption of normality. First, to examine differences in general and clinical characteristics across DII groups (low, intermediate, and high), non-parametric tests were employed: Mann–Whitney U and Kruskal–Wallis tests. Second, exploratory factor analysis with varimax rotation was conducted to identify the underlying nutrient clusters based on the dietary intake data. Factor scores derived from exploratory factor analysis were calculated for each participant. The exploratory factor analysis was conducted to explore patterns of co-consumed nutrients at the meal level; however, given the small number of participants, the resulting factor structure should be interpreted as exploratory and may partly reflect within-person dietary variability rather than stable between-person dietary patterns.

Third, to examine the overall associations between the nutrient cluster scores and E-DII tertiles, an ordinal logistic regression model with a logit link function was applied, as this approach appropriately accounted for the ordered nature of the dependent variables (low, intermediate, and high). Participants were categorised into these three groups based on tertile boundaries derived from the sample distribution of the total E-DII scores. Nutrient factor scores were simultaneously entered as independent variables. The proportional odds assumption was assessed and met, supporting the use of a single cumulative model. Odds ratios (ORs) and 95% confidence intervals (CIs) were estimated to quantify the likelihood of belonging to a higher E-DII tertile as a function of each nutrient cluster. Model fit and discrimination were evaluated using likelihood ratio statistics and concordance indices (C-statistics). Although regression was conducted at the meal level (*n* = 408), these records originated from only 17 individual participants, and no clustering adjustment was applied. Thus, the results should be interpreted as exploratory associations rather than individual-level predictions.

## 3. Results

Data from a total of 5 male and 12 female participants were analysed (Table 1). The mean age was 45.80 ± 11.48 years for males and 48.92 ± 12.34 years for females. Both groups had a mean BMI of approximately 28 kg/m^2^. Fasting glucose levels were 126.80 ± 22.17 mg/dL for males and 138.83 ± 31.98 mg/dL for females, and mean daily glucose levels were 126.10 ± 25.48 mg/dL and 144.60 ± 38.87 mg/dL, respectively. Homeostasis Model Assessment-Insulin Resistance values were 3.34 ± 2.57 for males and 4.41 ± 2.71 for females. Males showed higher triglyceride levels, whereas other lipid indicators were comparable between groups. Mean E-DII values were slightly negative in both groups. Although 408 meal-level observations were included in the final analysis, it is important to note that these data originated from only 17 participants, limiting the representativeness of the sample.

Five nutrient clusters were derived using principal component analysis with varimax rotation, accounting for 69.3% of the total variance (Table 2). The first cluster (labelled antioxidant-mineral) included dietary fiber, folic acid, Vitamin C, magnesium, and iron. The second cluster (i.e., the protein-B complex) comprises animal lipids, proteins, niacin, selenium, riboflavin, and thiamine. The third cluster (i.e., fatty acids) included SFAs, MUFAs, n-6 PUFAs, and n-3 PUFAs, with a reliability of 0.71. The fourth cluster (i.e., plant-lipids) comprised vegetable lipids and Vitamin E (Cronbach’s α = 0.74), and the fifth cluster (i.e., immune-modulating micronutrients) included pyridoxine and zinc (Cronbach’s α = 0.72). These five clusters represented dominant co-consumption patterns among nutrients with potential relevance to inflammation.

**Figure 1:**
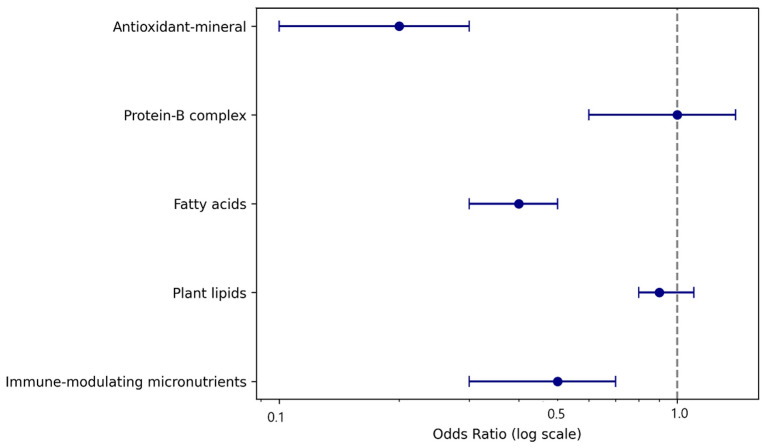
Forest plot of odds ratios for predicting E-DII tertiles.

An ordinal logistic regression analysis was conducted to identify nutrient cluster predictors of the E-DII tertiles (Table 3). Three nutrient clusters (antioxidant-mineral, fatty acids, and immune-modulating micronutrients) were significant predictors, each showing negative associations with higher E-DII levels. The model showed modest discrimination (C-statistic = 0.43), indicating limited classification ability; however, consistent directional effects were observed. The forest plot illustrating the ORs and 95% CIs for each nutrient cluster is shown in Figure 1. These results provide preliminary evidence of associations between nutrient intake patterns and dietary inflammatory potential across E-DII levels.

## 4. Discussion

This study aimed to identify nutrient clusters based on E-DII scores in patients with T2DM and prediabetes, and to construct exploratory models that classify these scores into E-DII tertiles. We identified five distinct nutrient clusters: antioxidant-mineral, protein-B complex, fatty acids, plant-lipids, and immune-modulating micronutrients, which reflect the underlying dietary patterns that contribute to E-DII variability. Although the clusters were derived from co-ingestion patterns rather than predefined biological categories, interpretations were made cautiously based on dominant nutrient effects and supported by previous literature. The labels were not intended to imply uniform mechanistic action, but rather to assist in describing the nutritional themes emerging from factor loadings. Furthermore, the classification models effectively distinguished participants into low, intermediate, and high E-DII tertiles. Higher intake of antioxidant-mineral, fatty acids, and immune-modulating micronutrient clusters was significantly associated with a lower probability of being in the high E-DII group.

Nutrient cluster 1, labelled ‘antioxidant–mineral,’ comprised dietary fibre, folic acid, Vitamin C, magnesium, iron, and carbohydrate. These nutrients are associated with antioxidant capacity and metabolic support. Magnesium and iron are not direct antioxidants; however, they serve as essential cofactors for antioxidant enzymes [26]. Moreover, magnesium intake has been inversely associated with inflammatory markers such as high sensitivity C-reactive protein, interleukin-6 (IL-6), and tumour necrosis factor-alpha (TNF-α) [27], whereas folic acid, Vitamin C, and iron contribute to immune function, oxidative stress regulation, and maintenance of arterial and renal health [28]. Therefore, they are believed to be grouped as mineral factors with similar antioxidant and immune activities.

Nutrient cluster 2, named ‘protein–B complex,’ included animal lipids, protein, niacin, riboflavin, thiamine, and selenium. Animal lipids are not micronutrients per se; however, their inclusion in the ‘protein–B complex’ cluster likely reflected shared dietary sources. Animal lipids and proteins are essential amino acids that support tissue repair and immune resilience, whereas B vitamins (particularly thiamine, riboflavin, and niacin) are crucial for coenzyme synthesis, energy metabolism, and cellular homeostasis. B vitamins and selenium support cellular metabolism, nicotinamide adenine dinucleotide or nicotinamide adenine dinucleotide phosphate synthesis, and antioxidant enzyme synthesis, while also playing roles in molecular-level immune regulation [29]. Some overlap existed with cluster 1 regarding antioxidant and immune-related functions; however, this cluster likely emerged independently because of its emphasis on energy metabolism and coenzyme function, rather than direct antioxidant or anti-inflammatory action.

Nutrient cluster 3, labelled ‘fatty acids,’ included SFAs, MUFAs, n-6 PUFAs, and n-3 PUFAs, which exhibit divergent physiological effects. SFAs and n-6 PUFAs are associated with pro-inflammatory responses and elevated low-density lipoprotein cholesterol levels, whereas MUFAs and n-3 PUFAs are known for their anti-inflammatory and cardioprotective benefits, including a reduced risk of metabolic syndrome and cardiovascular disease [30,31]. However, emerging evidence also highlights beneficial effects on n-6 PUFAs, such as cholesterol-lowering and improved glucose metabolism [32]. The clustering of these contrasting fatty acids likely reflected co-ingestion in habitual diets rather than shared biological mechanisms. Notably, the inclusion of SFAs may have been due to their high co-loading with MUFAs and PUFAs, indicating overlapping dietary sources. Thus, this cluster may represent general fat intake behavior rather than a biologically cohesive group with uniform inflammatory effects. Further research is needed to clarify the way in which co-intake patterns relate to inflammation and metabolic outcomes.

Nutrient cluster 4, composed of vegetable-based lipids and Vitamin E, reflected a dietary pattern rich in plant-derived fats from vegetable oils, nuts, and seeds. This cluster was associated with favourable metabolic profiles. Lower proportions of plant-derived fatty acids (e.g., linoleic and alpha-linolenic acids) have been linked to poorer glucose metabolism and a higher incidence of T2DM [33]. Vitamin E, commonly co-consumed with these fats, may further support metabolic health by reducing oxidative stress and proinflammatory cytokines (e.g., TNF-α and IL-6) through immune and membrane-stabilising functions [34]. Together, this cluster likely represents adherence to minimally processed, plant-based fat intake associated with improved insulin sensitivity and reduced cardiometabolic risk.

Pyridoxine and zinc were grouped into a distinct ‘immune-modulating micronutrient’ cluster due to their anti-inflammatory roles. Pyridoxine belongs to the B-complex family; however, it exerts unique immunological effects. High-dose pyridoxine downregulates pro-inflammatory mediators (including IL-1β, IL-6, TNF-α, and the NLR family pyrin domain containing three inflammasome components) in lipopolysaccharide-stimulated monocytes, indicative of broad-spectrum anti-inflammatory action [35]. Similarly, zinc supports both innate and adaptive immunity by boosting T-cell function, natural killer cell activity, and mucosal barrier integrity [36]. Their co-loading reflects both shared dietary sources and overlapping immunoregulatory effects, justifying their grouping in inflammation-focused dietary analysis.

Ordinary logistic regression revealed that the antioxidant-mineral cluster—comprising Vitamin C, folate, magnesium, and iron—significantly reduced the probability of belonging to the high E-DII group. These nutrients, mainly sourced from citrus fruits, leafy vegetables, legumes, and whole grains [37], emerged as a key differentiator of lower dietary inflammatory profiles. This finding aligns with prior research indicating an inverse association between total dietary antioxidant capacity and obesity-related traits in patients with T2DM [38]. Antioxidant-rich dietary patterns are known to support metabolic health and prevent abdominal obesity, which is highly prevalent among patients with T2DM. As such, encouraging the intake of foods high in antioxidant minerals may represent a practical and evidence-based strategy to reduce E-DII and mitigate inflammation-associated metabolic risks in this population.

The relationship between the fatty acids included in the cluster (i.e., SFAs, PUFAs, and MUFAs)—and T2DM or prediabetes remains somewhat inconsistent. MUFAs and PUFAs show inverse associations with T2DM and prediabetes [39]; however, other studies have revealed that MUFAs increased the risk of T2DM, whereas PUFAs and n-3 PUFAs reduced the risk [40]. This apparent inconsistency in associations may be partially explained by the fact that fatty acids are often co-consumed in mixed dietary sources, such as oils and animal products, making it difficult to isolate their individual effects. Still, consistent evidence indicates that SFA increase the risk of T2DM [40] and that n-3 PUFAs are positively associated with prediabetes [41]. Nevertheless, at the level of dietary intake patterns, MUFAs, n-3 PUFAs, and n-6 PUFAs may have exerted dominant effects in lowering the E-DII. This supports the interpretation of this cluster as a reflection of habitual fat intake rather than a unified biological mechanism. Promoting foods rich in unsaturated fatty acids (e.g., olive oil, avocados, nuts, and oily fish) may help to lower the E-DII and improve metabolic health. A recent review further recommended regular n-3 PUFA intake (e.g., fatty fish twice a week) for patients with T2DM, due to consistent anti-inflammatory and cardiometabolic benefits [42].

Pyridoxine has shown an inverse association with T2DM [43], and low serum levels have been linked to increased all-cause mortality in patients with T2DM [44], underscoring its clinical relevance. Zinc improves glucose control and insulin resistance in patients with prediabetes [45], with serum levels showing a linear relationship with T2DM and prediabetes risk [46]. Zinc supplementation reduces fasting glucose, insulin resistance, and glycated haemoglobin levels [47], and moderate dietary intake is associated with a 13% lower T2DM risk [48]. These findings support adequate, not minimal, intake of both nutrients. Common food sources include poultry, seafood, legumes, and whole grains. Beyond supplementation, regular intake of these micronutrients may help suppress inflammation, support immune function, and lower the E-DII, thus contributing to improved metabolic health.

Although the ordinal logistic regression models demonstrated statistically significant associations between nutrient clusters and E-DII tertiles, the discriminative performance was modest, with a C-statistic of 0.43, indicating a level below the generally accepted threshold for effective classification (typically ≥ 0.7). This suggests that while the models captured consistent directional relationships, their utility for individual-level prediction remains limited. From a statistical perspective, the low C-statistic reflected the complexity and variability of dietary patterns, as well as potential residual confounding. Clinically, this distinction is essential: the current model should not be interpreted as a screening or diagnostic tool, but rather as an exploratory framework to understand the way in which nutrient co-intake patterns relate to dietary inflammatory potential. By focusing on association rather than prediction, this study highlights dietary features that may inform nutritional counselling without overextending the applicability of the model.

Contrary to our expectations, the protein-B complex cluster was not a significant predictor of E-DII tertiles. However, the biological relevance of protein-B complexes should not be overlooked. Animal-based foods such as meat, fish, and dairy products provide abundant protein, B vitamins, and selenium [49], and a higher intake of these micronutrients has been linked to reduced metabolic and inflammatory risk [50]. Additionally, traditional Korean diets include diverse soy-based fermented foods such as soybean paste, soy sauce, and pepper paste (gochujang), which supply B vitamins and bioactive compounds with anti-inflammatory and metabolic benefits [51,52]. Collectively, dietary patterns that balance animal and plant protein sources, particularly through the inclusion of fermented soy products, may exert complementary effects in lowering dietary inflammatory potential.

### 4.1. Strength and Limitations

A key strength of this study is that while the dependent variable was the E-DII score, the independent variable was the intake of individual DII-related nutrients. Because the E-DII is a weighted index derived from literature-based inflammatory effect scores, our approach enabled direct assessment of actual nutrient intake patterns, thereby enhancing the clinical relevance of the findings for dietary counselling and intervention planning. However, this study had some limitations. Moreover, the small sample size from a single tertiary hospital limits the generalizability of the results, and the findings should be interpreted with caution when applied to broader populations. Considering the low discriminative power of the models, as reflected by the C-statistic, caution is warranted in extrapolating these findings beyond the current sample. Future studies with larger and more diverse populations are warranted to validate these nutrient clusters and to refine their utility in classifying dietary inflammatory potential across varying contexts. Replication using longitudinal or multi-site data could enhance the generalisability and potential translational value of these exploratory models.

In addition, multiple meal-level dietary records were obtained from the same patients, which may have introduced pseudo-replication and within-subject correlation. Although the analyses focused on exploratory associations at the dietary pattern level, these dependencies may have influenced variance estimation and should be addressed using multilevel or mixed effects approaches in future studies. Given the limited sample size and potential intra-individual correlations, the precise prediction of E-DII tertiles was inherently constrained. This analytical structure limited causal or predictive inference but remained appropriate for the exploratory aim of the present—study, i.e., to identify nutrient clusters that characterised lower or higher dietary inflammatory potential. Nevertheless, the observed relationships remained robust, indicating that the identified nutrient clusters reliably reflected underlying dietary inflammatory potential. Future studies should address these limitations by incorporating longitudinal dietary data and exploring the interactions between nutrient clusters and clinical outcomes, which may help clarify causal pathways and strengthen the translational value of E-DII-based models.

### 4.2. Implications for Clinical Nutrition

This study highlighted the importance of recognising nutrient clusters, rather than individual nutrients, when designing dietary strategies for patients with T2DM and prediabetes. Antioxidant-mineral, fatty acids, and immune-modulating micronutrients all demonstrated protective associations with lower E-DII levels, suggesting their potential role in reducing dietary inflammation. Therefore, clinicians and dietitians should encourage a balanced intake of fruits, vegetables, legumes, nuts, fish, and fermented soy products that provide these clusters. This approach may facilitate more personalised dietary counselling and help patients adopt sustainable eating patterns that lower inflammatory risk. Ultimately, translating cluster-based insights into practical food-based recommendations could enhance the effectiveness of nutritional interventions in metabolic care.

## 5. Conclusions

This study identified five nutrient clusters underlying E-DII variability and demonstrated their associations with pro- and anti-inflammatory dietary profiles in patients with T2DM and prediabetes. Antioxidant-mineral, fatty acid, and immune-modulating micronutrient clusters emerged as key factors distinguishing lower or intermediate E-DII groups from higher E-DII groups, underscoring their protective potential. These findings support the value of focusing on nutrient clusters rather than isolated nutrients in both research and clinical practice. While limitations such as model fit variability and repeated dietary records must be considered, the study provides a foundation for future studies exploring longitudinal dietary patterns and clinical outcomes. Ultimately, incorporating nutrient cluster-based models into dietary counselling may improve metabolic health and reduce the inflammatory burden in at-risk patients.

## Figures and Tables

**Table 1:** Participant Characteristics.

Characteristics	Categories	Male (*N* = 5)	Female (*N* = 12)
		n (%) or M ± SD	
Age (years)		45.80 ± 11.48	48.92 ± 12.34
Waist circumference (cm)		97.80 ± 8.11	93.95 ± 7.47
BMI (kg/m^2^)		28.78 ± 3.18	28.36 ± 4.20
	25–30	3 (60.0)	6 (50.0)
	≥30	2 (40.0)	6 (50.0)
Fasting glucose (mg/dL)		126.80 ± 22.17	138.83 ± 31.98
HbAlc (%)		6.84 ± 1.48	7.03 ± 1.06
	<6.5	2 (40.0)	6 (50)
	≥6.5	3 (60.0)	6 (50)
Insulin Resistance (HOMA-IR)		3.34 ± 2.57	4.41 ± 2.71
	<2.5	3 (60.0)	3 (25.0)
	≥2.5	2 (40.0)	9 (75.0)
Total Cholesterol		141.80 ± 25.11	150.08 ± 46.81
Triglyceride		191.00 ± 90.03	134.83 ± 38.91
HDL		46.58 ± 18.31	49.04 ± 7.70
LDL		76.20 ± 14.58	83.25 ± 35.64
Hypertension		1 (20.0)	1 (8.3)
Dyslipidemia		2 (40.0)	5 (41.7)
both		1 (20.0)	2 (16.7)
None		1 (20.0)	4 (33.3)
Diabetes Diagnosis	Pre Diabetes	2 (40.0)	5 (41.7)
	Diabetes	3 (60.0)	7 (58.3)
Treatment type	OHA	4 (80.0)	5 (41.7)
	OHA and insulin	1 (20.0)	7 (58.3)
E-DII per meal (Range: −2.00–1.89)		−0.109 ± 1.162	−0.011 ± 1.149
E-DII daily (Range: −2.50–2.41)		−0.241 ± 1.51	0.038 ± 1.39
Peak Postprandial Glucose per each meal (Range: 136.25–278.83)		189.85 ± 32.61	206.24 ± 46.62
Mean average glucose per day (Range: 100.46–207.82)		126.10 ± 25.48	144.60 ± 38.87

M ± SD = Mean ± Standard Deviation; BMI = Body Mass Index; HbA1c = Glycated hemoglobin; HOMA-IR = Homeostasis Model Assessment-Insulin Resistance; HDL = High Density Lipoproteins; LDL = Low Density Lipoproteins; OHA = Oral Hypoglycemic Agent; E-DII = Energy adjusted Dietary Inflammatory Index.

**Table 2:** Nutrient Intake and Factor-derived Nutrient Clusters.

Symptom	Intake (M ± SD)	Nutrient Clusters				
		Antioxidant-Mineral	Protein-B Complex	Fatty Acids	Plant-Lipids	Immune-Modulating Micronutrient
Diet fiber (g)	7.22 ± 4.85	0.769				
Folic acid (μg)	165.60 ± 121.19	0.759				
Vitamin C (mg)	28.09 ± 31.43	0.755				
Magnesium (mg)	37.94 ± 32.47	0.709				
Iron (mg)	6.17 ± 6.48	0.535				
Carbohydrate (g)	72.78 ± 27.08	0.363				
Animal lipids (g)	7.79 ± 10.39		0.858			
Protein (g)	23.40 ± 15.02		0.848			
Niacin (mg)	4.31 ± 3.74		0.789			
Selenium (μg)	21.41 ± 19.53		0.787			
Riboflavin (mg) (Vitamin B2)	0.46 ± 0.29		0.579			
Thiamine (mg) (Vitamin B1)	0.55 ± 0.34		0.501			
SFA (g)	3.09 ± 4.09			0.884		
MUFA (g)	4.21 ± 5.47			0.846		
N-6 PUFA (g)	1.26 ± 2.43			0.593		
N-3 PUFA (g)	0.22 ± 0.54			0.542		
Vegetable lipids (g)	7.84 ± 6.74				0.820	
Vitamin E (mg)	5.76 ± 4.82				0.696	
Pyridoxine (mg)(Vitamin B6)	0.69 ± 1.43					0.932
Zinc (mg)	3.30 ± 2.37					0.747
Eigen value		3.84	3.78	2.39	2.07	1.79
Cumulative variance explained (%)		19.18	38.09	50.02	60.35	69.32
Cronbach’s alpha		0.60	0.73	0.71	0.74	0.77

M ± SD = Mean ± Standard Deviation; SFA = Saturated Fatty Acids; MUFA = Monounsaturated Fatty Acids; N-6 PUFA = Omega-6 Polyunsaturated Fatty Acids; N-3 PUF = Omega-3 Polyunsaturated Fatty Acids.

**Table 3:** Risk prediction models for dietary inflammatory index levels using ordinary logistic regression (*N* = 408).

Variable	Estimate	SE	z	*p*-Value	OR	95% CI
Antioxidant-mineral	−2.47	0.21	−12.02	<0.001	0.08	0.06–0.13
Protein-B complex	−0.08	0.11	−0.74	0.459	0.92	0.75–1.14
Fatty acids	−0.78	0.14	−5.65	<0.001	0.46	0.35–0.60
Plant-Lipids	−0.09	0.11	−0.77	0.442	0.92	0.74–1.14
Immune-modulating micronutrient	−1.07	0.26	−4.10	<0.001	0.34	0.21–0.57

SE = standard error; OR= odds ratio; CI= confidence intervals.

## Data Availability

The original contributions presented in this study are included in the article/Supplementary Material. Further inquiries can be directed to the corresponding author.

## Supplementary Materials

The following supporting information can be downloaded at: https://www.mdpi.com/article/10.3390/nu18030422/s1, Table S1: STROBE Statement—Checklist of items that should be included in reports of cross-sectional studies.

## Abbreviations

DII	Dietary inflammatory index
E-DII	Energy-adjusted dietary inflammatory index
SFA	Saturated fatty acids
MUFA	Monounsaturated fatty acid
T2DM	Type 2 diabetes mellitus
n-3 PUFA	Omega-3 fatty acids
n-6 PUFA	Omega-6 fatty acids
ICC	Intraclass correlation coefficient
ORs	Odds ratio
CIs	Confidence intervals
TNF-α	Tumour necrosis factor-alpha
IL-6	Interleukin 6
